# Angiographic Pulse Wave Coherence in the Human Brain

**DOI:** 10.3389/fbioe.2022.873530

**Published:** 2022-05-03

**Authors:** Matthew J. Koch, Phan Q. Duy, Benjamin L. Grannan, Aman B. Patel, Scott B. Raymond, Pankaj K. Agarwalla, Kristopher T. Kahle, William E. Butler

**Affiliations:** ^1^ Department of Neurosurgery, University of Florida, Gainesville, FL, United States; ^2^ Medical Scientist Training Program, Yale University School of Medicine, New Haven, CT, United States; ^3^ Department of Neuroscience, Yale University School of Medicine, New Haven, CT, United States; ^4^ Department of Neurosurgery, Yale University School of Medicine, New Haven, CT, United States; ^5^ Department of Neurosurgery, University of Washington Medicine, Seattle, WA, United States; ^6^ Department of Neurosurgery, Massachusetts General Hospital, Boston, MA, United States; ^7^ Department of Radiology, University of Vermont, Burlington, VT, United States; ^8^ Department of Neurosurgery, Rutgers New Jersey Medical School, Newark, NJ, United States; ^9^ Division of Genetics and Genomics, Boston Children’s Hospital, Boston, MA, United States; ^10^ Broad Institute of MIT and Harvard, Cambridge, MA, United States; ^11^ MGH Hydrocephalus and Neurodevelopmental Disorders Program, Massachusetts General Hospital, Boston, MA, United States

**Keywords:** angiography, hydrocephalus, biomechanics, pulse waves, cerebral circulation

## Abstract

A stroke volume of arterial blood that arrives to the brain housed in the rigid cranium must be matched over the cardiac cycle by an equivalent volume of ejected venous blood. We hypothesize that the brain maintains this equilibrium by organizing coherent arterial and venous pulse waves. To test this hypothesis, we applied wavelet computational methods to diagnostic cerebral angiograms in four human patients, permitting the capture and analysis of cardiac frequency phenomena from fluoroscopic images acquired at faster than cardiac rate. We found that the cardiac frequency reciprocal phase of a small region of interest (ROI) in a named artery predicts venous anatomy pixel-wise and that the predicted pixels reconstitute venous bolus passage timing. Likewise, a small ROI in a named vein predicts arterial anatomy and arterial bolus passage timing. The predicted arterial and venous pixel groups maintain phase complementarity across the bolus travel. We thus establish a novel computational method to analyze vascular pulse waves from minimally invasive cerebral angiograms and provide the first direct evidence of arteriovenous coupling in the intact human brain. This phenomenon of arteriovenous coupling may be a physiologic mechanism for how the brain precisely maintains mechanical equilibrium against volume displacement and kinetic energy transfer resulting from cyclical deformations with each heartbeat. The study also paves the way to study deranged arteriovenous coupling as an underappreciated pathophysiologic disturbance in a myriad of neurological pathologies linked by mechanical disequilibrium.

## Introduction

The brain is not an inert organ that simply resides motionless inside the cranium. As early as 1700 BC, ancient Egyptians had already recognized that the brain is a pulsatile organ (Goodrich, 2018). Thousands of years later, Volcher Coiter (1534–76) associated mechanical pulsations of the brain with arterial pulses generated by the heart (Goodrich, 2018).

It is now well-understood that pulsatile brain motion is a consequence of blood moving in and out of the cerebrovascular tree driven by pumping of the heart. Despite taking up only two percent of the total body weight, the brain receives up to one-fifth of the total cardiac output with every heart beat (Williams and Leggett, 1989). This means that ∼20 ml of blood arrives to the brain with each heart beat given a typical cardiac stroke volume of ∼100 ml, yet an intracranial hematoma at a volume as little as 10 ml may suffice to be fatal (Leasure et al., 2019). Thus the brain receives a quantity of blood that is greater than what might be fatal if it were merely deposited, suggesting the presence of physiological constraints that allow the brain to survive the immense mechanical forces resulting from dynamic in and outflow of blood (Wilson, 2016). These physiological constraints are likely stricter in the brain compared to peripheral tissues given that the brain resides within a rigid bony compartment (Wagshul et al., 2011). This housing of the brain inside a rigid compartment has given rise to the Monro-Kellie doctrine, which holds that the intracranial cavity contains a fixed sum volume of blood, brain, and cerebrospinal fluid (CSF), and an interplay between these three components generates the intracranial pressure (ICP) (Wilson, 2016). A change in any of the three intracranial components should produce reciprocal changes in the other two. The physiological mechanisms that permit proper dispensation of volume and energy transfer with each heartbeat to maintain mechanical equilibrium remain poorly understood. Furthermore, stroke volume and pulse wave shape are regulated by multiple mechanisms (neural activity, respiratory movement, and body movement) with a strong cerebral autoregulation (Liu et al., 2020a; Claassen et al., 2021). The interplay between physical forces and physiological regulation inside the cranium is an area of active investigation.

At present, much of our understanding regarding intracranial pulsatility (which encompasses movement of brain tissue, CSF, and blood) has been driven by the use of three techniques to measure pulsations: ICP monitoring, transcranial Doppler ultrasound (TCD), and magnetic resonance imaging (MRI) (Wagshul et al., 2011). Despite the widespread use of these methods in research and clinical practice, they are inadequate to fully characterize brain pulsations for multiple reasons. First, while ICP monitoring and TCD have high temporal resolution to register single cardiac frequency events, they provide limited spatial information of the entire intracranial system. An additional disadvantage of ICP monitoring is that it is often invasive. Second, while MRI is noninvasive, it does not have sufficient temporal resolution to adequately characterize cardiac frequency phenomena in the absence of critical assumptions. MRI imaging of cardiac frequency phenomena in the brain typically relies on cardiac gating (Bhadelia et al., 1997; Kim et al., 1998; Kachelriess et al., 2000; Markl et al., 2012), in which the timing of image acquisition is matched to an external cardiac signal (typically an electrocardiogram) to enable reconstruction of images within an averaged cardiac cycle (Bernstein et al., 2004; Butler, 2017). Rather than directly imaging events internal to a given cardiac cycle, cardiac gating assumes that all heartbeats are alike and thus are not able to capture phenomena that differ between each heartbeat. In order to adequately image events that occur/oscillate at cardiac frequency (such as intracranial pulsations) without losing information, the sampling rate of the imaging method must be faster than that of the cardiac frequency as per the sampling theorem of Kotelnikov, Nyquist, and Shannon (Nyquist, 1928; Vladimir Aleksandrovich Kotelnikov, 1933; Shannon, 1949). Current techniques to study intracranial pulsatility are thus too invasive or do not have sufficient sampling rate in order to satisfactorily interpret the complex interplay of cerebrovascular physiology that occurs at cardiac frequency.

In previous work, we demonstrated using a piglet open cranial window model and human open cranial data the utility of wavelet transformation in evaluating the interplay between cardiac pulsations and cerebral perfusion (Butler, 2017; Butler et al., 2017). Wavelet transformation is a powerful mathematical tool used in signal processing that permits the capture of very minute details and sudden changes in the raw signals, acting as a “mathematical microscope” to examine properties of the signals that may be hidden or otherwise difficult to appreciate (Faust et al., 2015). A distinct advantage of wavelet transformation is that it reveals what frequencies are present in the signal in a spatiotemporal context whereas a standard Fourier transform reveals only what frequencies are present in the signal and thus would not be able to show when in time the signal has changed. We applied wavelet transformation to the analysis of angiographic data acquired faster than cardiac rate as per the Nyquist sampling theorem (Nyquist, 1928; Vladimir Aleksandrovich Kotelnikov, 1933; Shannon, 1949) in order to produce cardiac frequency phenomena images in a one to one relation from a source angiogram, permitting cardiac frequency activity to be reconstructed frame by frame from within a single contrast injection. We found that wavelet transformation of angiographic data was able to resolve individual arterial and venous pulse waves in the intact piglet brain and in human open cranial windows (Butler, 2017), suggesting the utility of wavelet transformation as a mathematical tool to characterize intracranial pulsatility from images acquired at faster than cardiac rate.

In this study, we apply a similar wavelet transformation methodology to human cerebral angiography to evaluate whether intracranial cerebrovascular physiology can be evaluated with minimally invasive imaging techniques. X-ray cerebral angiography is a clinical diagnostic procedure that does not require direct surgical cranial exposure and may be employed for quantitative cerebral blood flow analysis. Images are typically acquired at faster than the human cardiac rate of about 1–1*.*5 Hz. We ask whether a similar cross-correlated wavelet reconstruction applied to human x-ray angiograms could permit the resolution of individual vascular pulse waves in the brain. If so, we further ask whether these vascular pulse waves may be classified into arterial and venous, and whether they are reciprocally coherent.

We hypothesize that the brain organizes its circulation such that an arriving arterial stroke volume produces an arterial pulse wave upon its entry into the cranial vault, and that this is compensated by a reciprocally coherent venous pulse wave. In this model, the existence of reciprocal coherence between the brain’s arterial and venous subsystems permits the intracranial compartment’s total blood volume to remain relatively constant across the cardiac cycle. We pursue two experimental predictions. If a brain artery can be identified by anatomy in an angiogram, its pixels should pulse with shared phase and the venous anatomy should be the aggregation of pixels in the angiogram pulsing with reciprocal phase. Conversely, if a brain vein can be identified by anatomy, its pixels should pulse with shared phase and the arterial anatomy should be the aggregation of pixels pulsing with reciprocal phase. If the above experimental predictions can be satisfied, we may conclude that the brain’s circulation contains reciprocally coherent arterial and venous subsystems, providing a physiologic explanation for how the brain maintain a state of mechanical equilibrium in the setting of dynamic in and outflow of blood associated with each heartbeat. Understanding these mechanisms not only yields insights into fundamental cerebrovascular physiology of the brain, but also has important diagnostic, prognostic, and treatment implications for a wide myriad of neurological disorders or pathological states linked by abnormal brain pulsatility, mechanical functions, and cerebrovascular coupling (Wagshul et al., 2011; Park et al., 2012; Butler, 2017; Butler et al., 2017; Eide, 2021).

## Results

**FIGURE 1:**
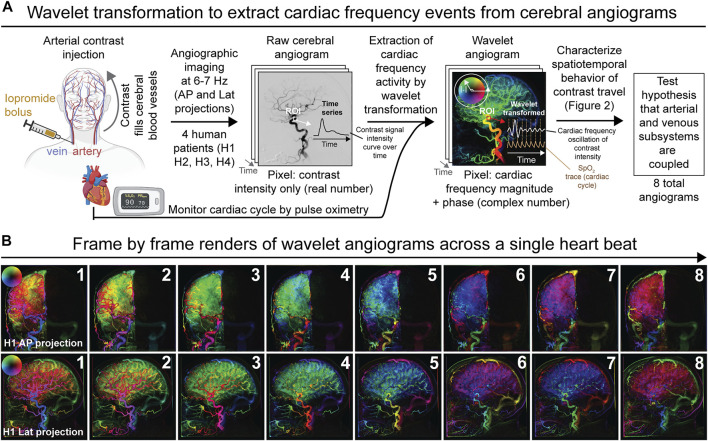
Overview of experimental and analytical approach to analyze diagnostic cerebral angiograms by wavelet transformation. **(A)** Schematic depicting the derivation of wavelet angiograms from raw cerebral angiograms acquired in neurologically unremarkable human patients. In summary, the wavelet transformation permits extraction of contrast intensity signals that oscillate at cardiac frequency from the raw cerebral angiograms. Analysis of the wavelet transformed data from different anatomical components of the cerebrovascular network allowed us to test the hypothesis that the arterial and venous subsystems are coupled. **(B)** Wavelet transformed right common carotid angiogram during the duration of a single heartbeat demonstrating multiple spatiotemporal phase groupings for subject H1 (frame rate 6.0 Hz). Both anterior-posterior (AP) and lateral (lat) projections are shown.

The overall methodological approach for our study and main findings are summarized in Figure 1A and Supplementary Video S1.

To begin, we performed routine cerebral transfemoral angiography from four neurologically unremarkable human subjects (referred to as subjects H1, H2, H3, and H4, Supplementary Table S1 and methods). We first injected a bolus of intra-arterial iodinated contrast into the arterial system and sequentially imaged contrast travel throughout the cerebrovascular tree by non-invasive X-ray angiography acquired at a rate of 6–7 Hz, which is substantially faster than the normal cardiac frequency of 1–2 Hz. The sampling rate of the X-ray angiography thus satisfies the Nyquist sampling theorem with regards to detecting signals that change at cardiac frequency. A different artery was injected for each of the four subjects and the imaging was acquired in two dimensions (anterior-posterior or lateral projections) for each subject, yielding a total of eight raw cerebral angiograms for the study (see Supplementary Video S1 for an example of raw cerebral angiogram acquired from a subject). Viewing the raw cerebral angiogram shows the sequential filling of the tracer first in the arterial systems then the veins, demonstrating the expected pattern of blood in and outflow from the brain. Thus, tracer distribution is used as a proxy of blood circulation and cerebral perfusion. While acquiring the cerebral angiograms, cardiac activity was also monitored for all subjects by pulse oximetry.

At baseline, the raw cerebral angiogram is essentially a simple time-series depicting changes in the contrast signal intensity across time. To characterize the interplay between cardiac pulsations and cerebral perfusion, we extracted contrast intensity signal changes that oscillate at cardiac frequency from the cerebral angiograms. We applied wavelet transforms to the pixel-wise time signals from the raw angiograms and filtered the transformed data for cardiac wavelet scale from the external cardiac signals, thereby generating a wavelet transformed angiogram also termed the pulse wave angiogram (Figures 1A,B). The wavelet angiogram has the same number of pixels and frames as the raw angiogram but every pixel is now represented by a complex number, which may be represented in polar form wherein magnitude signifies cardiac frequency magnitude and phase represents cardiac frequency phase. For rendering, magnitude is represented as brightness and phase as hue, starting in the positive horizontal direction and rotating counterclockwise as per the circular arrow (Figures 1A,B). This permits the representation of cardiac frequency activity as on the example wavelet angiograms (Figure 1B for example wavelet angiograms acquired from subject H1 and Supplementary Figure S1 for wavelet angiograms acquired from all other subjects). The computational validation of this wavelet method against simulated angiograms with known noise and known cardiac frequency behavior is presented in Supplementary Figure S2, wherein we show that the wavelet methods can recover cardiac frequency activity mixed with noise in simulated angiograms. Thus, by cross-correlating angiographic data (signal intensity of the injected contrast across time) with an external cardiac signal, our wavelet algorithm was able to extract cardiac frequency phenomena from the raw cerebral angiograms. Qualitative examination of direct frame by frame renderings of the wavelet transformed angiogram for a single heartbeat selected from the middle of the bolus travel showed surprisingly complex patterns of cardiac frequency activity (Figure 1B and Supplementary Figure S1).

**FIGURE 2:**
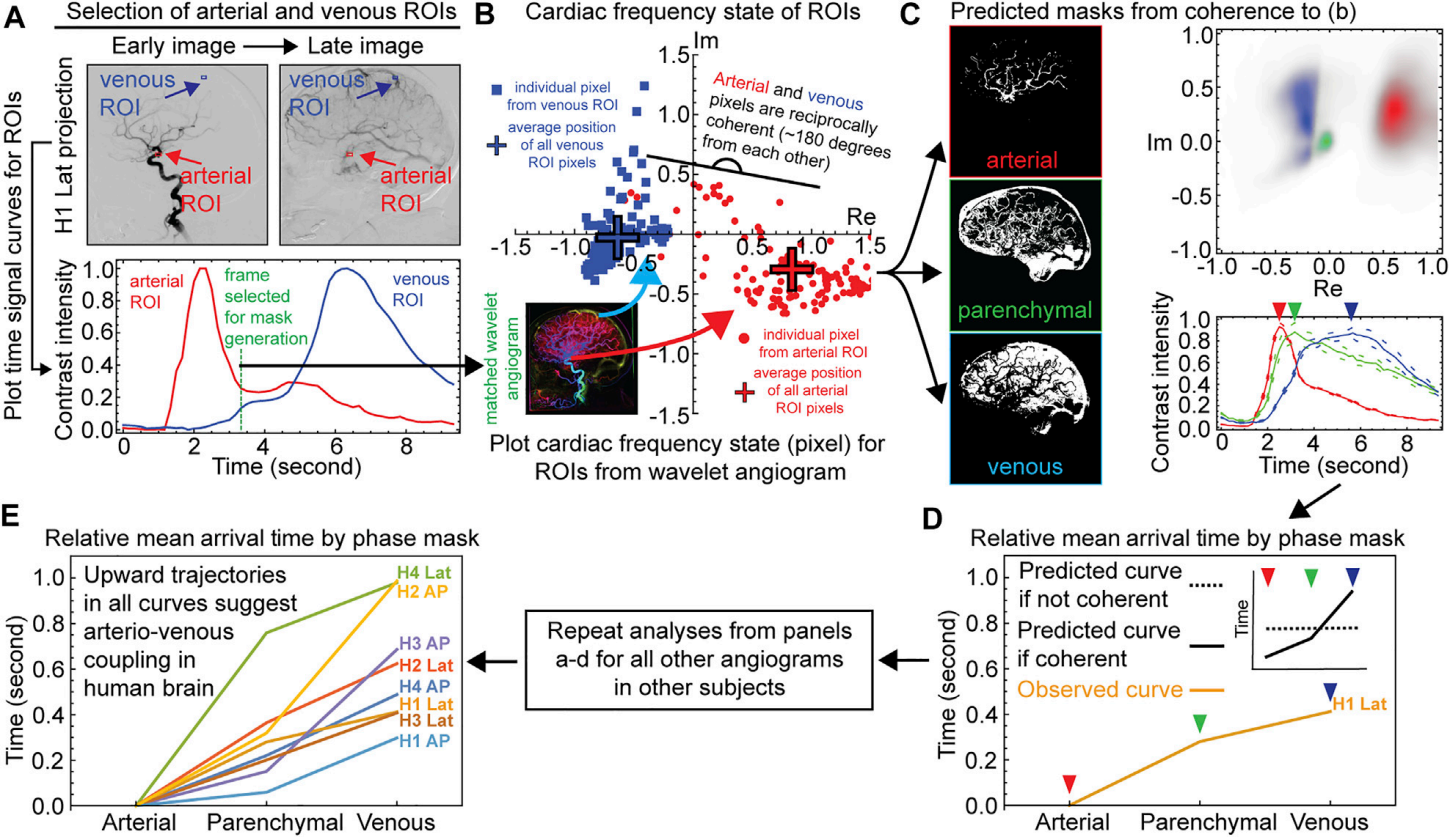
Demonstration of reciprocal coherence between arterial and venous systems of the human brain. Example data for one angiographic projection from subject H1 are shown for panels a–c, data from all other projections are shown in Supplementary Figures S3, S4 Named Vessel ROIs, Right Common Carotid Artery Distribution, Lateral Projection. The top left shows angiographic frames in arterial and venous phases of the bolus travel with named artery and vein ROIs. The bottom left shows time signal curves for the two ROIs. The vertical gray bar indicates the frame selected for mask generation. The right panel shows complex-valued scattergrams for the two ROIs after wavelet transformation for cardiac frequency. Frame rate 6.0 Hz. Subject H1. c) Time Signal Curves From Coherence Masking, Right Common Carotid Artery Distribution, Lateral Projection. The left column shows the arterial, parenchymal, and venous coherence masks as per the named artery and vein ROIs in panel a. The top right shows complex-valued histograms of the wavelet-transformed cardiac frequency data for the masks. The bottom right shows angiographic time intensity curves generated from the masks are given with standard error. d-e) Relative mean arrival times from coherence masking for each subject, AP and lateral projections.

We next characterized coupling of vascular pulse waves between the arterial and venous subsystems from the set of eight wavelet angiograms from the four subjects. The results for the lateral projection in the common carotid artery from subject H1 is shown Figure 2 while analyses for the other seven angiograms are shown in Supplementary Figures S3, S4. We selected a putative artery and vein by visual inspection of each angiogram and placed a small region of interest (ROI) in it (Figure 2A). We confirmed the respective arterial and venous identity by reviewing the time signal curves showing changes in the contrast intensity in the respective ROI across time (Figure 2A and Supplementary Figure S3). An arterial ROI should have an early bolus passage and a vein ROI a late one, and indeed the time signal curves for the arterial ROI showed an early peak whereas the venous ROI showed a later peak (Figure 2A and Supplementary Figure S3). We then selected a frame (time coordinate) between the arterial and venous peaks, and within this frame we analyzed the cardiac frequency activity of the arterial and venous ROI from the wavelet angiogram (Figure 2A and Supplementary Figure S3). We plotted the complex numbers represented by individual pixels from the two ROI on a scattergram and found that pixels from the arterial and venous ROIs were nearly reciprocal to each other (different by 180 degrees), and these findings were consistent across all wavelet angiograms analyzed for all subjects (Figure 2B and Supplementary Figure S3).

Given that respective vessel ROIs were sufficient to predict each other’s phase, we asked whether the information within the small ROIs could be generalized to predict the global cerebrovascular tree anatomy inside the cranium. We generated arterial or venous spatial coherence masks by gathering all pixels that are coherent to the named arterial and venous ROI, respectively (Figure 2C and Supplementary Figure S4, methods). We also generated an intermediate parenchymal mask from pixel members of the intracranial mask not in the arterial or venous masks (Figure 2C and Supplementary Figure S4). Complex-valued scattergrams of the wavelet-transformed cardiac frequency data for the masks again demonstrated reciprocal coherence between the predicted arteries and veins (Figure 2C and Supplementary Figure S4). To confirm that the globalized coherence masks indeed reflect their predicted vascular anatomy (arterial vs. venous subsystems), we applied the masks to the entire raw space angiogram to generate time signal curves of contrast travel (Figure 2C and Supplementary Figure S4). These time signal curves demonstrated relative bolus travel timing consistent with representing arterial, parenchymal, and venous beds Figure 2C and Supplementary Figure S4). All angiographic studies display the ordinal relation where the arterial precedes the parenchymal precedes the venous coherence mask mean arrival time (Figures 2D,E and Supplementary Figure S4). Under the null hypothesis, this is approximately a one in 6^8^ or 1*.*68 *×* 10^6^ events.

## Discussion

We introduce and computationally validate a wavelet method of directly imaging cardiac frequency activity from fluoroscopic angiograms acquired at faster than cardiac rate. This allows us to test the hypothesis within a single fluoroscopic angiographic sequence that an arriving arterial stroke volume induces an arterial pulse wave in the brain that it accommodates with a reciprocally coherent venous pulse wave. We find that the pulse phase selected from a single frame in a small named artery ROI contains sufficient information to predict pixel-wise recognizable arterial anatomy and its reciprocal pulse phase contains sufficient information to predict recognizable venous anatomy. When applied as masks to the entire raw space angiogram, the predicted arterial and venous images generate time signal curves with relative arterial and venous bolus passage timing. Conversely, the pulse phase and its reciprocal phase selected from a single frame in a small named vein ROI respectively predict arterial and venous anatomy sufficiently to be recognized in images and to generate time signal curves with arterial and venous bolus passage timing.

Furthermore, the predicted arterial and venous pixel groups maintain a complementary phase difference across the bolus travel. By definition, when two signals are coherent, their phase is coupled such that knowing the phase of one enables the inference of the phase of the other. The capacity to infer the properties of the arterial subsystem from the venous subsystem and vice-versa supports the hypothesis of arteriovenous pulse wave coherence. We employ cardiac frequency phase from a single time coordinate in a named vessel ROI to predict the relative arteriovenous position of brain angiographic time signal curves across the entire time range of the bolus passage. This is a measure of the consistency of coherence within and between heartbeats.

**FIGURE 3:**
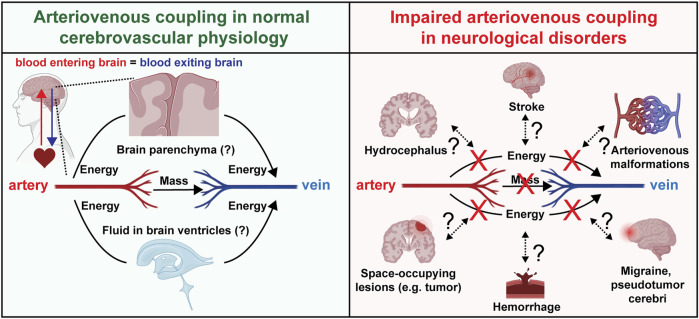
Arteriovenous coupling in normal physiology and neurological disorders. Schematic depicting hypotheses regarding arteriovenous coupling in normal cerebrovascular physiology and potential involvement in multiple human neurological disorders.

The maintenance of coherence between the arterial and venous subsystems implies the exchange of phase information between them (Figure 3A). It appears unlikely that this occurs endoluminally through the capillaries because experimental cranial window microscopy studies have not found red blood cells in brain capillaries to have pulsatile velocity changes (Unekawa et al., 2010). Alternatively, the phase coupling information may be exchanged across the intervening extravascular tissue by cyclical deformations at cardiac frequency. Such a mechanism would depend on the biomechanical properties of the intervening brain tissue and cerebrospinal fluid reservoirs (Goriely et al., 2015; Butler et al., 2017) (Figure 3A).

Clinically, the establishment of this physiology and computational methods to evaluate it as presented in this paper permits future investigations to gain a more nuanced understanding of a multitude of pathologies afflicting the brain. Abnormal intracranial pulsatility has been proposed to be involved in numerous neuropathological conditions with or without elevated ICP (Wagshul et al., 2011; Park et al., 2012; Butler, 2017; Butler et al., 2017; Eide, 2021). The potential role of arteriovenous coupling in maintaining the brain’s mechanical equilibrium motivates future studies to investigate how this apparent coherence may be disrupted in various conditions in which brain circulation is disrupted directly (e.g., ischemic and hemorrhagic strokes, migraines, arteriovenous malformations) or indirectly (e.g., space-occupying lesions) (Figure 3B). Impaired arteriovenous coupling may lead to abnormal exchange of kinetic energy between the arterial and venous systems, leading to dysregulated dissipation of energy and volume with each heartbeat. This impairs the brain’s endogenous capacity to absorb the “shock” that comes with an arriving arterial stroke volume, thus exposing the brain to “cardiogenic” trauma.

Noninvasive computational tools, such as wavelet transformed angiograms, to monitor perturbations in arteriovenous coupling may permit the identification of novel physiologic markers of mechanical disequilibrium that would enable to clinician to intervene in due time to avert catastrophic sequelae such as rapidly fatal brain herniation. In addition to mean arrival time as analyzed by this study, other parameters of the vascular waveform may be extracted to fit the multidimensional models of cerebral blood flow (Liu et al., 2020b) in which arteriovenous coupling can be added to existing models consisting of ICP, arteries, and veins. These parameters may include the slope and location of the maximal first derivative. Given that diagnostic cerebral angiograms are safe and ubiquitously acquired as part of the standard diagnostic investigation for patients with neurological complaints, the computational methods we introduce to extract cardiac frequency activity from angiograms may be retrospectively applied to pre-existing angiograms to investigate novel aspects of cerebrovascular physiology.

Our study has several limitations that motivate continued study in future investigations. First, we note that the sampling frequency should be higher than twice the cardiac frequency to satisfy the Nyquist-Shannon sampling theorem. However, this covers only the fundamental harmonic. Studies in photoplethysmography pulse wave have shown that even a threshold of 5 Hz in low-past filtering may produce inaccuracies in the waveform (Allen and Murray, 2004; Liu et al., 2021a), including deformation of the waveform and changes in the locations of feature points. These limitations could be addressed by a sampling frequency of 7 Hz, which may detect higher (>2) harmonics more reliably. Given that some of the angiograms in this study were acquired at 6 Hz, there may be some inaccuracies in the detected pulse wave. Second, our sample size is currently limited to four patients harboring incidentally discovered cerebral aneurysm, which may itself influence cerebral hemodynamics. Indeed, cerebral blood flow has complex regulatory mechanisms (Liu et al., 2020a; Claassen et al., 2021), and many physiological factors such as blood rheology, mental stress, and atherosclerosis may influence cerebral blood flow patterns (Wang et al., 2005; Liu et al., 2021b). These considerations motivate further validation of our findings in normal subjects as well as human patients harboring a variety of pathophysiological conditions.

In summary, wavelet reconstructions of sequential angiographic image frames identify individual traveling vascular pulse waves. The organization of these pulse waves suggests that the brain hosts a complex secondary cardiac cycle that includes coherence between the arterial and venous subsystems. This is consistent with the finding of arteriovenous phase locking in brain ultrasound and optical angiography (Butler, 2017). Further work is needed to furnish a hypothetical framework for analyzing them. However, wavelet angiographic methods such as these that can resolve single vascular pulse waves may complement the scope of testable hypotheses that is offered by cardiac-gated imaging methods (Sagel et al., 1977; Bhadelia et al., 1995; Kim et al., 1998; Kachelriess et al., 2000; Mistretta, 2011; Markl et al., 2012)^.^ Ultimately, our work paves the way for future investigations to characterize cerebrovascular physiology in the live intact human brain and to generate a comprehensive spatiotemporal “atlas” of arteriovenous coupling in physiologic and pathologic states.

## Methods

### Subjects

With approval from our institutional IRB, with written consent, we performed angiogram analysis from four human subjects undergoing routine cerebral transfemoral angiography (Supplementary Table S1). These were pre-treatment angiograms of a previously diagnosed incidentally found cerebral aneurysm. As a consequence, these are not comprehensive diagnostic angiograms with contrast injections into the four major vessels (right and left carotid and vertebral arteries), but are limited to one major vessel of interest. The subjects have normal neurological exams and do not have headache on the days of these studies. These are not angiograms performed during a hospitalization for diagnosis or treatment of an acute cerebrovascular condition such as stroke or aneurysmal subarachnoid hemorrhage. Hence we believe that the cerebrovascular physiology demonstrated in the angiograms is normal or near normal. None of the subjects dropped out. The clinical procedure has a favorable safety profile (Dawkins et al., 2007). Its application was not altered by participation in this study.

### Cerebral Angiography

Diagnostic cerebral angiography was performed on an Artis Zee biplane (Siemens, Washington DC) using native images gathered at a rate ranging from 6 to 7 frames per second. This angiographic unit is capable of faster frame acquisition rates but we do not employ them because our approval for use of human subjects specifies that we adhere to our routine practices. A bolus of intra-arterial iodinated contrast, iopromide (Bayer, Berlin, Germany) was injected by manual control of a syringe. The contrast bolus volume was up to 10 ml. The injection rate was at the discretion of the operator and not controlled across angiograms. The biplane system produces two angiographic image projections per contrast bolus injection, anteroposterior (AP) and lateral. Prior to the diagnostic angiogram, the operator injects a small quantity of contrast to verify catheter position, the position of the biplane fluoroscopy unit, and vessel integrity. The mean radiation dose area product for the entire angiographic study (not just the run analyzed here) was 155 ± 22 *Gy cm* ^2^. This falls within the common range for cerebral angiography (Ihn et al., 2016).

Concurrent noninvasive physiologic data consisting of an electrocardiogram, pulse oximetry, and automated manometric blood pressure were obtained as a routine aspect of anesthesia care. The vital signs including heart rate and blood pressure remained within the normal human range. These were recorded in synchrony with angiographic imaging with an integrated audiovisual recording system (Black Diamond Video, Steris Inc., Mentor, Ohio, United States). The audiovisual recording system simultaneously captures the physiological traces and the angiographic images as they are produced. The pulse oximeter trace in particular is employed as a cardiac signal cross-correlated in subsequent wavelet analysis of the angiographic data. The images retained for wavelet analysis were downloaded in DICOM format from the hospital image archive and de-identified.

### Wavelet Transformation To Extract Cardiac Frequency Activity

We extracted the cardiac frequency activity from angiograms using a previously established wavelet algorithm that spatiotemporally cross-correlates angiographic phenomena with an external cardiac signal (Butler, 2017). The method requires that the angiographic data be acquired at faster than cardiac rate as per the sampling theorem of Kotelnikov, Nyqvist, and Shannon (Nyquist, 1928; Vladimir Aleksandrovich Kotelnikov, 1933; Shannon, 1949). This requirement is satisfied by these human cerebral angiograms.

To summarize, we used Gabor wavelets to filter cardiac frequency phenomena in the angiographic images. A separate cardiac signal is measured from an optical plethysmogram on the patient’s skin. A Gabor wavelet filter is applied to the external cardiac signal using comparatively high frequency resolution. We extract from this a cardiac frequency filtered cardiac signal that we save for use as a cardiac signal cross correlated. Each pixel on the angiographic image sequence is then treated as an independent time signal. It is Gabor wavelet transformed and in wavelet domain each pixel time signal is cross-correlated to the cardiac signal cross correlated. The result is inverse Gabor wavelet transformed into time domain. Each pixel of each frame is then represented by a complex number that represents cardiac frequency magnitude and phase. The polar form of a complex number may be rendered pixel-wise in an image with a brightness-hue color model as illustrated in Figure 1A. All calculations were performed in the Mathematica version 11 environment (Wolfram Research, Urbana, Illinois, United States).

### Coherence Criteria From Named ROIs

After wavelet transformation, each frame is a pixel by pixel complex-valued representation of cardiac frequency magnitude and phase. The real and imaginary components in a given vessel both oscillate at cardiac frequency. The relation between the real and imaginary components gives the cardiac frequency phase. The cardiac frequency activity in the named artery and vein ROIs is analyzed from the transformed angiogram. Within each ROI the cardiac frequency activity is coherent because it is a uniform population of blood. There is a polar representation for a complex number where there is a magnitude and a phase angle. If two pixels are in the same half arc (within +*/− π/*2 deg or +*/−* 90 deg of each other) then we term them as relatively coherent. If not, then they are reciprocally coherent. This is calculated arithmetically for a pixel by multiplying its complex conjugate by the mean wavelet transformed value in an ROI. If the result is positive, then they are coherent, if negative then they are reciprocally coherent.

For each angiographic projection, we select a frame (time coordinate) between the early time signal peak in the arterial ROI and the later time signal peak of the venous ROI. We find that the cardiac frequency phase in the named arterial ROI is nearly reciprocal (different by 180 deg) to the cardiac frequency phase in the named venous ROI. We ask if this reciprocal coherence pattern in a named artery and vein can generate a valid pixel-wise prediction of arterial and venous anatomy throughout the entire angiogram. We find the same result regardless of which frame we employ for analysis. For convenience, we select in each case a frame corresponding to a peak in the real component in the arterial ROI of the wavelet-transformed angiogram. The named ROIs and their time signal curves are shown in Figure 2A and Supplementary Figure S3.

### Spatial Coherence Masks

We create by hand an outer mask that contains only intracranial pixels. To create an arterial coherence mask, we gather all pixels that are coherent to the named arterial ROI and binarize them using Otsu’s method (Bangare et al., 2015). This is not a simple thresholding method but instead uses a spatial correlation criterion as part of the binarization. As a consequence, Otsu’s method tends to bring out confluently coherent structures such as blood vessels while excluding coherent single pixels without coherent neighbors. The intersection of these pixels with the intracranial mask is taken to be the arterial mask.

A similar method is employed to generate the venous mask. Otsu’s binarization method is applied to those pixels coherent to the named venous ROI, and the intersection of these with the intracranial mask is taken to be the venous mask. An intermediate parenchymal blush mask is generated from the pixel members of the intracranial mask that are neither in the arterial nor the venous masks. Pixels in the named arterial and venous ROIs are excluded from the masks to prevent circular arguments.

### Time Signal Curves

We generate time signal curves by summing the pixel values for each angiographic image frame after application of a mask. In the plots, the arterial, parenchymal, and venous time signal curves are separately scaled linearly. To estimate angiographic signal error for a given mask, at each frame we compute a mean and standard deviation signal intensity of the masked values. The masks have significantly different numbers of pixels. We normalize for mask pixel size by reporting the data as mean ± standard error.

### Mean Arrival Time

To compare the central tendency of the time signal curves we compute the mean arrival time of each. This is equivalent to computing the mean value of the time axis weighted by the height of the time signal curve.

$$\bar{t}=\frac{\sum_{i=1}^{n}s_{i}t_{i}}{\sum_{i=1}^{n}s_{i}}$$

The curves are generated by pixel masks according to cardiac frequency coherence to named artery and vein ROIs. This method tends to underestimate the mean arrival time for a mask where there remains residual contrast agent after the last angiographic frame.

Under the null hypothesis of no organized coherence, then the arterial coherence mask, parenchymal mask, and venous mask mean arrival times should have a random relation to each other. The study hypothesis predicts that the time signal curve mean arrival time for the arterial coherence mask should precede that of the parenchymal mask which should precede that of the venous mask. Since there are 3 coherence masks, there are 3! = 6 possible mean arrival time orderings per angiographic projection. Expanded across the subjects and projections, this gives 6^8^ *≈* 1*.*68 *×* 10^6^ ordering combinations, of which one corresponds to the study hypothesis where arterial coherence mask *<* parenchymal *<* venous mean arrival times. This process is illustrated in Supplementary Video S1.

### Monte Carlo Statistical Error

We checked this analysis with Monte Carlo simulations where for each angiographic projection we generate a random mask by randomly subsampling 100 pixels from each mask and producing a time signal curve. We repeated this 1000 times and examined the distribution of generated time signal curves. They correspond closely to the mean ± standard error values as described above.

### Computational Validation of Cardiac Frequency Phenomena Representation in Wavelet Transformed Angiography

We computationally validate the wavelet transformation method by measuring the statistical distributions of bolus passage and cardiac frequency parameters in limited ROIs in these angiograms. These parameter distributions are used to assign to pixels random but known bolus travel and cardiac frequency parameters. These parameter are employed to generate the sequential frames of an angiogram. The simulated angiogram is wavelet analyzed and the calculated cardiac frequency parameters are compared to the assigned ones statistically and by visual inspection.

The bolus travel is parameterized as a gamma distribution (Weiss, 1983; Barfett et al., 2014). It can skew the bolus contrast profile, for example to include a right skew when modeling the contrast time intensity curve of an arterial pixel.

The cardiac frequency parameters (that include the representation of cardiac frequency phase) are drawn from the arterial ROI are randomly placed into the arterial mask and vice versa for the venous parameters. The example simulated angiogram of Supplementary Figure S2 is derived from the data of subject H1. The ROIs are drawn from Figure 2C.

The bolus travel and cardiac frequency parameter distributions are employed to generate a simulated angiogram. We subject the simulated angiogram to wavelet cardiac frequency restoration to see if the arterial and venous masks can be resolved and the cardiac frequency parameters recovered. The gener-ation of simulated angiograms with cardiac frequency activity and the recovery of the cardiac frequency parameters by separate software modules shows that the wavelet restoration inverts the cardiac frequency simulation. This is shown for the example of subject H1 in Supplementary Figure S2. The visual inspection of the images with known cardiac frequency patterns and the wavelet-restored ones shows demonstrates recovery of the cardiac frequency parameters. This is reinforced by a pixel-wise analysis of the simulated and restored arterial and venous mask pixel values (Supplementary Figure S2H **)**.

In control simulations, the parameter distributions drawn from the arterial and venous ROIs are mixed so that the arterial and venous masks have no significant difference in cardiac frequency parameters. The wavelet transformation of these simulated angiograms do not show an arteriovenous difference in cardiac frequency phenomena.

## Data Availability

Our data are publicly available at https://doi.org/10.6084/m9.figshare.7080896.
