# Supplementary material for: Angiographic Pulse Wave Coherence in the Human Brain
Source: Front Bioeng Biotechnol. 2022 May 3;10:873530. doi: 10.3389/fbioe.2022.873530 (PMC9110661; doi:10.3389/fbioe.2022.873530)
Supplement: Supplementary file 1 [file DataSheet1.pdf]

SUPPLEMENTARY INFORMATION

Subject	Injected vessel	Aneurysm Segment	Frame Rate (Hz)	Subtraction
H1	Right Common Carotid Artery	Superior Hypophyseal	6	+
H2	Left Vertebral Artery	Basilar Apex	7	-
H3	Right Internal Carotid Artery	Ophthalmic	6	+
H4	Right Vertebral Artery	Left Cavernous	7	-

Supplementary Table 1 S1 (related to Figure 1): Information on all studied subjects, including vessels injected with contrast.

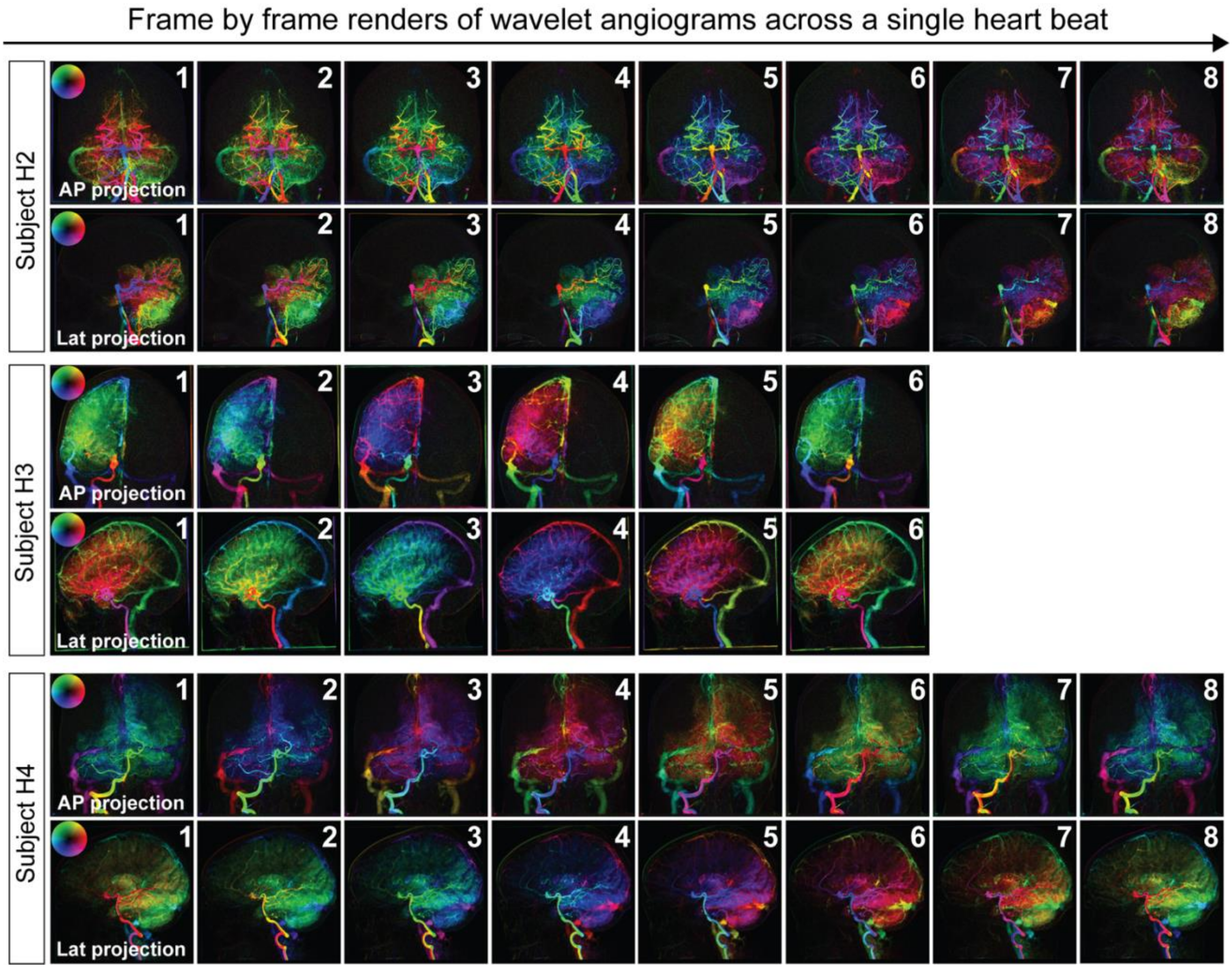

Supplementary Figure S1 (related to Figure 1): Wavelet transformed angiograms during the duration of a single heartbeat demonstrating multiple spatiotemporal phase groupings for subjects H2, H3, and H4. Both anterior-posterior (AP) and lateral (lat) projections are shown for each subject.

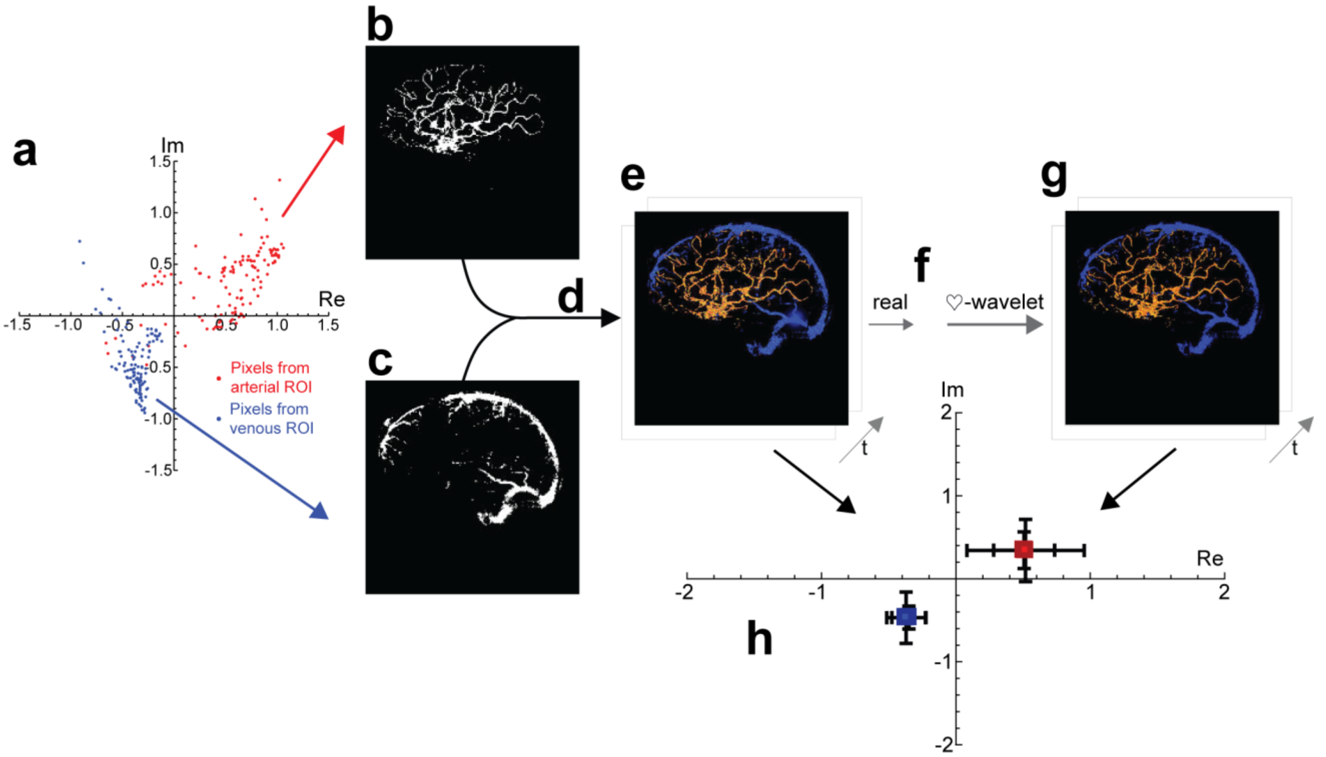

Supplementary Figure S2 (related to Figure 1): Simulated angiography with ♥-wavelet restoration. a) Separate distributions of arterial and venous cardiac frequency parameters are drawn from named arterial and venous ROIs from subject H1. The distributions are use to populate pixels in an (b) arterial mask and a (c) venous mask. The distributed parameters are (d) merged then used to drive cardiac frequency phenomena across the image frames of the entire angiographic sequence. e) An example image frame is rendered using the brightness-hue color model for cardiac space representation. f) To simulate angiography, the real component is extracted, then subjected to ♥-wavelet restoration as per the methods presented in this paper. g) The same frame of the restored data is rendered for comparison. The images are similar but not identical. h) The mean and SD complex value data for the arterial and venous masks in the simulated data and ♥-wavelet restored data are co-plotted to demonstrate the statistical correspondence.

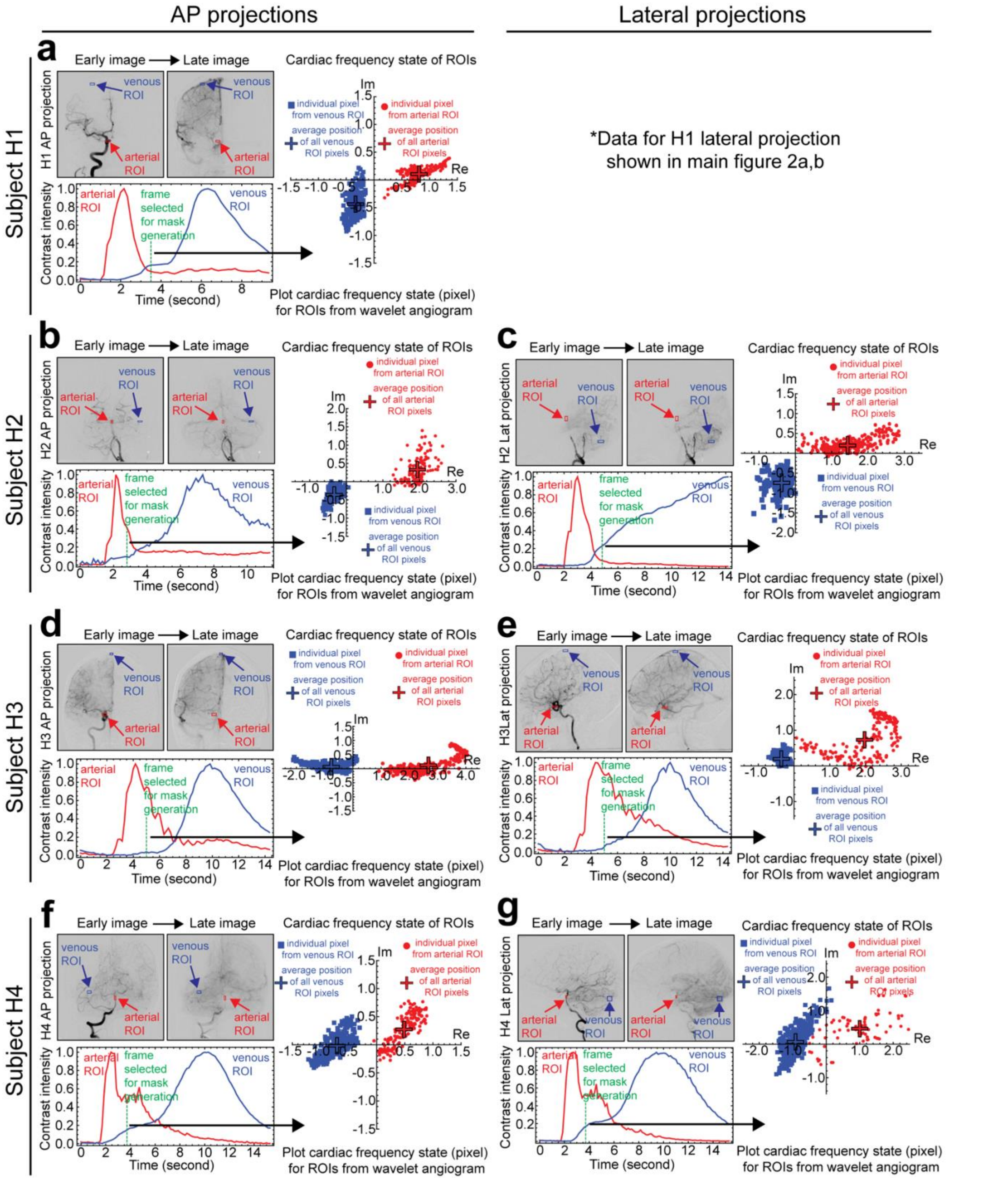

Supplementary Figure S3 (related to Figure 2): Named region of interest generation and analysis for all studied subjects. Panels a-g show data for each projection (anterior-posterior – AP, or lateral) from the corresponding subject. Data from the lateral projection angiogram of subject H1 are shown in main figure 2a-b. Within each panel, the top left shows angiographic frames in arterial and venous phases of the bolus travel with named artery and vein ROI. The bottom left shows time signal curves for the two ROI. The vertical green bar indicates the frame selected for further analysis and mask generation. The right panel shows complex-valued scattergrams for individuals pixels in the two ROI after wavelet transformation for cardiac frequency. The scattergrams from all projections showed that the pixels from the respective ROI (arterial or venous) were ~180 degrees of each other, indicating reciprocal coherence between the arteries and veins.

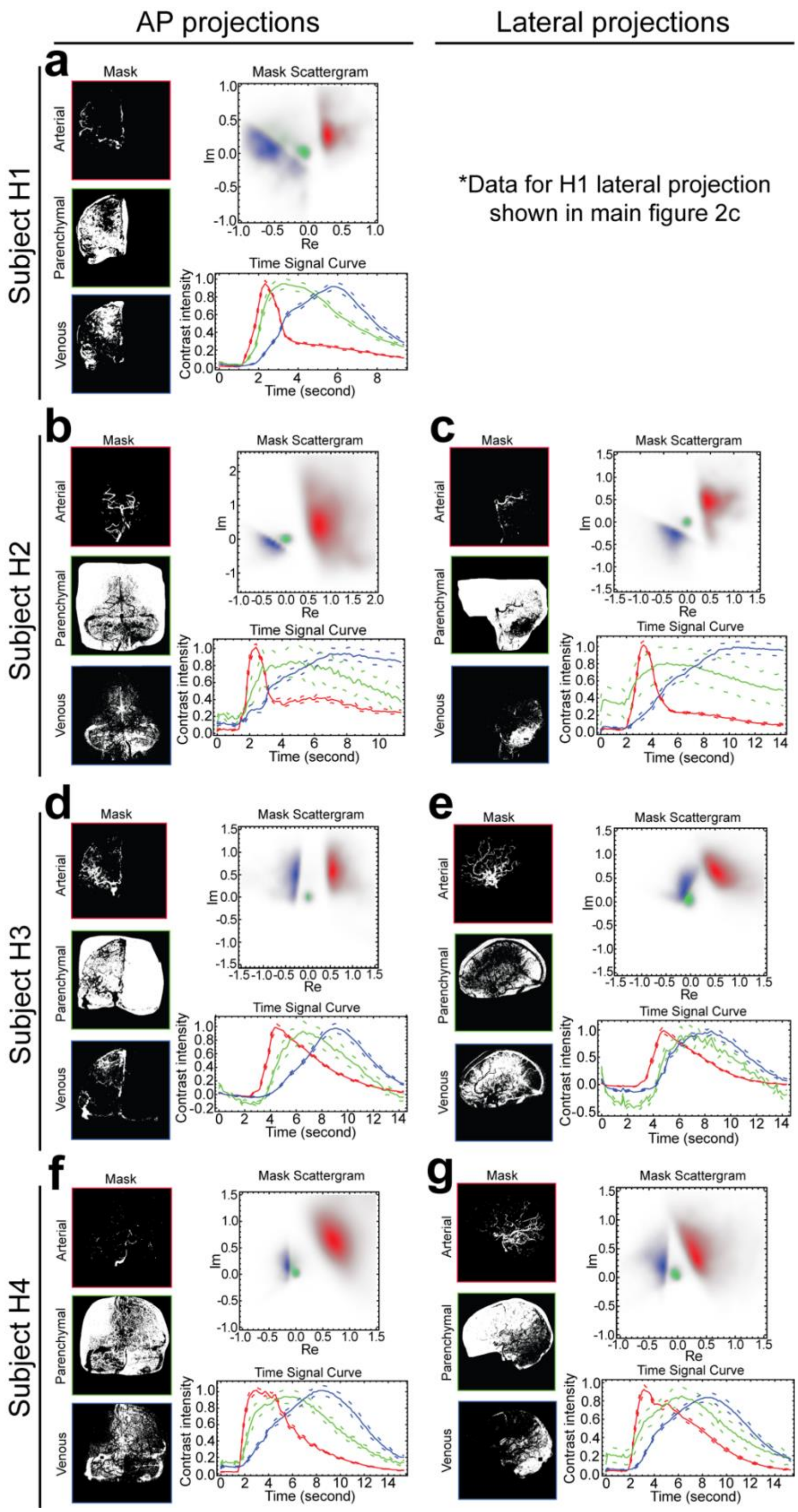

Supplementary Figure S4 (related to Figure 2): Arteriovenous coherence analyses for all studied subjects. Panels a-g show data for each projection (anterior-posterior – AP, or lateral) from the corresponding subject. Data from the lateral projection angiogram of subject H1 are shown in main figure 2c. The left column shows the arterial, parenchymal, and venous coherence masks as per the named artery and vein ROIs in Supplemental Figure 4. The top right shows complex-valued histograms of the wavelet-transformed cardiac frequency data for the masks. The bottom right shows angiographic time intensity curves generated from the masks (arterial is red, parenchymal is green, venous is blue) are given with standard error.

Supplementary Video 1 S1 (related to Figures 1 and 3): Summary of methodology and overall findings of the study. Demonstration of the methodology whereby images are obtained using diagnostic cerebral angiography, undergo wavelet transformation, and from here are further evaluated for arterial and venous wave forms. Then the generation of arterial and venous masks, mean arrival time, and ultimate error calculations are illustrated.
